# Gelatin-Coated Microfluidic Channels for 3D Microtissue Formation: On-Chip Production and Characterization

**DOI:** 10.3390/mi10040265

**Published:** 2019-04-19

**Authors:** Gabriele Pitingolo, Antoine Riaud, Claudio Nastruzzi, Valerie Taly

**Affiliations:** 1 INSERM UMR-S1147, CNRS SNC5014, Paris Descartes University, Equipe Labellisée Ligue Nationale Contre le Cancer, 75005 Paris, France; antoine.riaud@gmail.com; 2 Dipartimento di Scienze Chimiche e Farmaceutiche, Università di Ferrara, 44121 Ferrara, Italy; nas@unife.it

**Keywords:** 3D microtissues, microfluidics, human colon adenocarcinoma, cells viability, dehydration, gelatin, hydrogel

## Abstract

Traditional two-dimensional (2D) cell culture models are limited in their ability to reproduce human structures and functions. On the contrary, three-dimensional (3D) microtissues have the potential to permit the development of new cell-based assays as advanced in vitro models to test new drugs. Here, we report the use of a dehydrated gelatin film to promote tumor cells aggregation and 3D microtissue formation. The simple and stable gelatin coating represents an alternative to conventional and expensive materials like type I collagen, hyaluronic acid, or matrigel. The gelatin coating is biocompatible with several culture formats including microfluidic chips, as well as standard micro-well plates. It also enables long-term 3D cell culture and in situ monitoring of live/dead assays.

## 1. Introduction

For many years, cells cultured on flat monolayers have represented the reference in vitro models to grow and study cells and to evaluate the cellular mechanism in response to biophysical and biochemical stimulations. However, it has been described that two-dimensional (2D) culture conditions do not faithfully reflect in vivo conditions given that proper tissue architecture and cell–cell interactions are partially lacking in monolayers [1]. Three-dimensional (3D) cell cultures have therefore been introduced with the objective to better mimic in vivo systems compared to cells cultured as monolayers on plastic [2,3,4,5]. Recently, it was demonstrated that the gene expression profiles [6] as well as the responses to drug treatment in 3D tumor models resemble the in vivo situation more closely [7]. Therefore, 3D cell models, including the well-described production of 3D microtissues, are increasingly being employed as biologically relevant systems for drug development and preclinical drug testing [8]. Particularly, in the field of anticancer drug development, the use of 3D microtissues for drug testing appears appropriate. It is well known that tumor cells cultured in vitro with traditional models in conventional static 2D culture in multiwell plates poorly mimic the pathological environment associated with avascular tumors. In this respect, 3D microtissues are far better at reproducing inherent O_2_, nutrient, and metabolite gradients, providing a more accurate prediction of drug toxicity and efficacy.

Numerous 3D culture models currently exist for cancer-related research, such as liquid overlay-based culture [9], non-adhesive surfaces [10], stirred-tank culture strategies [11], and hanging drops [12]. However, many of these culture systems are characterized by a high labor cost or a limited capacity to mimic the extracellular matrix (ECM) [13]. In this respect, the 3D microtissues are often used in suspension (i.e., not attached to a substrate), making experiments in flow conditions difficult (i.e., to simulate blood flow). More recently, microfluidics has also been explored to obtain 3D tumor microtissues, often called spheroids [14,15]. Compared to traditional 3D cell culture methods, microfluidics possesses many advantages such as high-throughput and low-cost for drug screening. In addition, microfluidics also offers the possibility to work in flowing conditions for high-fidelity microtissue models and to integrate hydrogels to create biological microstructures [16].

With respect to the production of systems including ECM, recent studies have been performed using hydrogels to mimic native ECM [17] and as cell-instructive materials [18]. Hydrogels obtained with natural biopolymers have been employed to develop 3D cell culture systems and to create scaffolds for tissue engineering to mimic the in vivo microenvironments and cellular support matrices [19,20]. Hydrogels have also been integrated in microfluidic systems for tissue engineering application [21].

Biomaterials for 3D cell cultures are generally expensive and therefore not always usable within large screening platforms; they include collagen type I [22], hyaluronic acid [23], and Matrigel [24]. In this respect, gelatin could represent an interesting alternative to these costly biomaterials. Gelatin is a heterogeneous mixture of water-soluble proteins of high average molecular masses, present in collagen; it has been routinely used to modify substrates/matrices that are inert to cells [25]. Interestingly, gelatin forms thermoreversible gels, but to guarantee its use in biomedicine, chemical or physical cross-linking procedures are often needed [26]. Unfortunately, the chemical crosslinking protocol reactions (i.e., the residues of unreacted crosslinkers) can result in adverse cellular effects [27]. To cite some examples, Anseth et al. [28] and Khademhosseini et al. [29] proposed photo-crosslinkable gelatin hydrogels to promote 3D cell cluster formation of aortic valvular interstitial and HepG2 liver cancer cells, respectively. Both research groups prepared gelatin modified with methacrylamide (named GelMA) to create covalently stabilized gelatin hydrogels for in situ cell encapsulation and 3D cell culture. The GelMA hydrogel approach was also used by other authors to prepare stable hydrogels for modularly engineering biomimetic osteon and epidermal tissue engineering, respectively [30,31]. The majority of methods for 3D microtissue production with gelatin involve the use of rather complex processes and chemical crosslinkers, potentially resulting in toxic effects on the seeded cells.

An alternative cross-linking method for gelatin hydrogels is represented by controlled dehydration, as reported by Yannas et al. The authors demonstrated that gelatin becomes covalently cross-linked when the water content falls below a critical trace level of about 0.2 g/100 g protein [32]. Recently, this method was re-introduced to fabricate cross-linked gelatin molds with variable size (depending on the dehydration of the gelatin dispersion) to produce microfluidic chip platforms [33]. In addition, the gelatin cross-linking by dehydration was also employed to obtain the reversible bonding of different materials currently employed in microfabrication, such as poly(methyl methacrylate) (PMMA), PDMS, and glass (paper submitted). Interestingly, up to now, no articles have appeared, to the best of our knowledge, describing the production of 3D microtissues by gelatin substrates cross-linked by dehydration (i.e., without the use of chemical cross-linkers).

In the current paper, the use of the gelatin dehydration process is described to produce ultra-low attachment microfluidic channels for 3D microtissues with human colon adenocarcinoma (HT-29). Notably, the gelatin hydrogels were fabricated into a PMMA microfluidic chip and, as control on 6-well plates. To determine cell viability and proliferation rate, the generated 3D microtissues were analyzed using live/dead assay and live cells counting. Finally, to morphologically characterize the 3D microtissue in the proposed novel substrate, confocal microscopy analyzes were performed.

## 2. Materials and Methods

### 2.1. Materials and Equipment

The poly(methyl methacrylate) (PMMA) slabs used in this study, thickness 1.2 mm, were from a single batch purchased from Goodfellow Cambridge Ltd., (Huntingdon, UK). Gelatin from porcine skin type A was obtained from Sigma-Aldrich (St. Louis, MO, USA). The microfabrication process was performed by a micromilling, Minitech Machinery Corporation (Norcross, GA, USA), equipped with microtools (flute end mills diameter of 889 μm) from Performance Micro Tool (Janesville, WI, USA). Silicone peroxide tubing/60 Shore i.d. 0.75 mm obtained from IDEX Health & Science Gmbh (Oak Harbor, WA, USA) and Hypo Needles 18 AWG purchased from Warner Instruments LLC (Hamden, CT, USA) were used to test the microfluidic chip sealing.

### 2.2. Fabrication of Microfluidic Chips and Gelatin Coating

**Figure 1:**
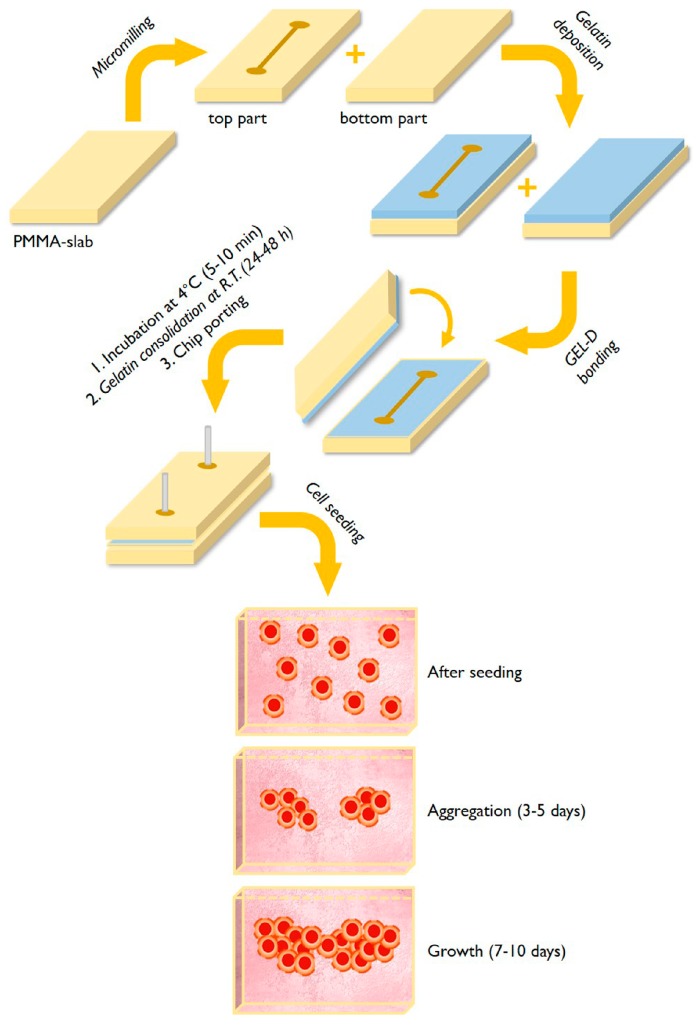
General scheme of the fabrication of gelatin-coated microchips (upper part) and representation of the cell’s aggregation and 3D microtissue formation (lower part).

Microfluidic chips were designed and fabricated using PMMA slabs (thickness 1.2 mm) by micromilling. For the micromachining, a CNC micromilling machine was employed. During micromilling, spindle speed, feed speed, and plunge rate per pass were set at 10,000 rpm, 15 mm∙s^−1^, and 20, respectively. The produced slabs have the following characteristics: (A) a top slab (75 mm × 20 mm, length/width) containing a linear 35 mm length microchannel (880/880 µm, width/depth) and (B) a bottom slab acting as sealing part for the microchip. The deposition of gelatin coating and chip sealing, named GEL-D bonding method (paper under revision), were performed following the general scheme reported in Figure 1. A gelatin dispersion in water (15% w/v), degassed under vacuum for 10 min to eliminate bubbles, was heated at 70 °C for 10 min. Typically, 500–800 µl of the warm (45–50 °C) gelatin dispersion in form of a droplet was deposited at the center of both the top and bottom PMMA parts of the chip; thereafter, both slabs were spun at high speed (1500 rpm for 20 s) using a spin coater (Laurell, WS-650 Series, North Wales, PA, USA) or, alternatively, a low-cost modified computer cooling fan, as recently reported [34]. The top and bottom parts of the chip were assembled, clamped and the sealed chip was incubated at 4 °C for 5–10 min and maintained at room temperature (24 °C) for further 24–48 h before use. The control uncoated chips were bonded by a solvent (ethanol) evaporation method [35].

### 2.3. Cell Seeding and Characterization

The in vitro cell culture experiments on gelatin-coated chips were performed using the HT-29 and HepG2 cell lines. Cells were purchased from American Type Culture Collection (ATCC, Manassas, VA, USA) and routinely cultivated in RPMI 1640 and DMEM media, respectively, supplemented with penicillin-streptomycin solution (10,000 U/mL) and 10% Fetal bovine serum (FBS). Before cell seeding, to prevent contamination, the microfluidic chips were treated with a 10% penicillin-streptomycin solution in Phosphate-Buffered Saline (PBS) for 12 h at 4°C. Cells were seeded in the microfluidic chips (50 µL, 5 × 10^4^ cells/mL) by a micropipette. The seeded microfluidic chip was maintained in a 5% humidified CO_2_ incubator at 37°C. Cells growth was monitored using inverted light microscope for up to 10 days. Pictures of the channel were taken after the seeding and at days 1, 2, 5, 7, 8, 9, and 10. Quantitative analyses of 3D tumor microtissue distribution and sizes (on chip) were performed using the ImageJ software (NIH, Bethesda, MD, USA). The surface area of the 3D microtissues was determined by tracing the contours with the freehand function in ImageJ and measured using the measure function. A surface area of 9 mm^2^ was analyzed for each experiment.

The live/dead staining was performed using Calcein AM and Sytox orange nucleic acid stains at concentrations of 1 μM and 0.250 μM, respectively, and cell death visualized with an epifluorescence microscope [36]. Hoechst 33342 (1 µL/mL) was used for nuclei staining. The 3D structure analyses were performed using a confocal microscope Leica equipped with 4× and 10× objectives. The cells were fixed using a solution of 2% Glutaraldehyde in 0.1M Phosphate Buffer, pH 7.3. After 20 min, the fixative agent was removed and the samples were washed by flowing three times PBS in the microchannel. After permeabilization with 0.5% Triton-X100, the cells were incubated with blocking buffer (1% bovine serum albumin (BSA) in PBS) and stained with Phalloidin for 1 h at room temperature and 4′,6-diamidino-2-phenylindole (DAPI) (1 µL/mL)

## 3. Results and Discussions

### 3.1. Fabrication of Gelatin-Coated Microfluidic Channels

The process for the fabrication of microchips with gelatin-coated microchannels is schematized in Figure 1. The microchips were produced with a micromachining approach using PMMA for both the top and bottom parts of the chip.

The microfluidic chips contain a simple linear 35 mm length microchannel (880/880 µm, width/depth); the fabrication process involves the deposition, on both the top and bottom parts of the microchip, of a small volume (500–800 µL) of a pre-melted (at 45–50 °C) gelatin dispersion in water by spin coating. In this respect, the gelatin layers formed by spin coating act both as coating for the microchannels and as bonding material for sealing the chip.

Notably, the produced chips were particularly robust in term of absence of leakage even if when the microchip are operated at high flow rates.

In order to have robust and reproducible results in terms of coating and bonding, it is important to could down at 4 °C for 5–10 min the freshly bonded chips; thereafter, the on-chip gelatin layer is left to slowly dehydrate (resulting in gelatin bonding, as described in the introduction). For 24–48 h at room temperature before the chip can be used for cell seeding.

The resulting deposited gelatin film on the microchannel was measured by profilometer, determining a thickness of about 6 µm.

### 3.2. Cells Aggregation and 3D Microtissue Formation

After dehydration (48 h), the chips were seeded with HT-29 or HepG2 cells by a micropipette. Twenty-four hours after seeding, differences in the adhesion and aggregation were analyzed between cells cultured in microchannel with or without gelatin coating and those cultured in conventional 6-well plates (used as control conditions).

**Figure 2:**
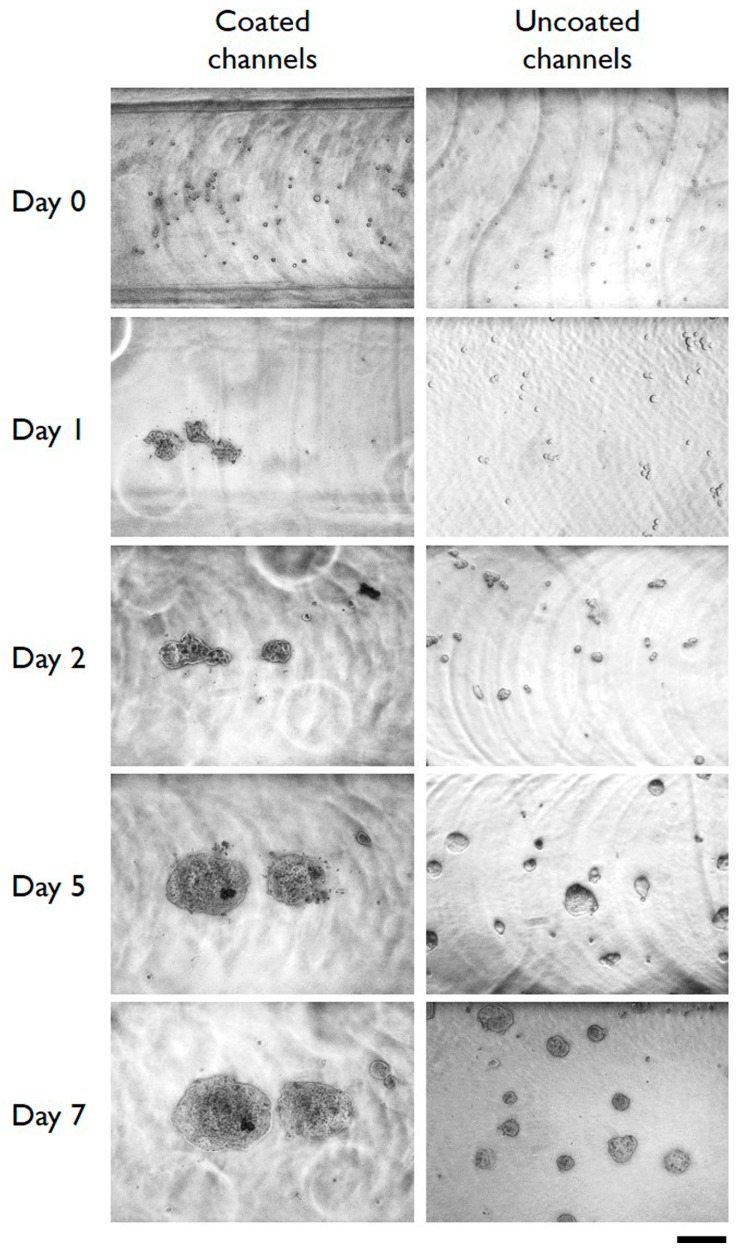
Typical microphotographs of HT29 cells cultured in gelatin-coated microchannels (left column) and in control uncoated microchannels (right column). Images were taken immediately after the seeding (day 0) and after the indicated length of in vitro cell culture. Scale bar: 200 µm. The depicted experiments were carried out in triplicate.

The results reported in Figure 2 and Figure S1 indicate that the cells cultured on gelatin-coated microchannels do not adhere to the substrate, whereas they have a strong tendency to form cell-to-cell contacts forming cell aggregates starting from day 1 of cell culture. The data reported in the supplementary material, relative to cells grown in conventional 6-wells plate, confirms that the cells seeded on gelatin film do not adhere (in form of single cells) to the substrate but rather form on the contrary cell aggregates. A notable difference between cells on chip and cells on plate resides in the fact that cells on chip form aggregates with an almost spherical shape; in addition, the 3D microtissues obtained in microfluidic conditions remain as single aggregate, for at least 6–7 days from the seeding, whereas the microtissues formed in wells start forming a graph structure from day 1–2. These data agree with those reported by Davidenko et al. that stated that gelatin thin film impedes the attachment of tumor cells to the culture substrate, promoting cell–cell adhesion [37].

**Figure 3:**
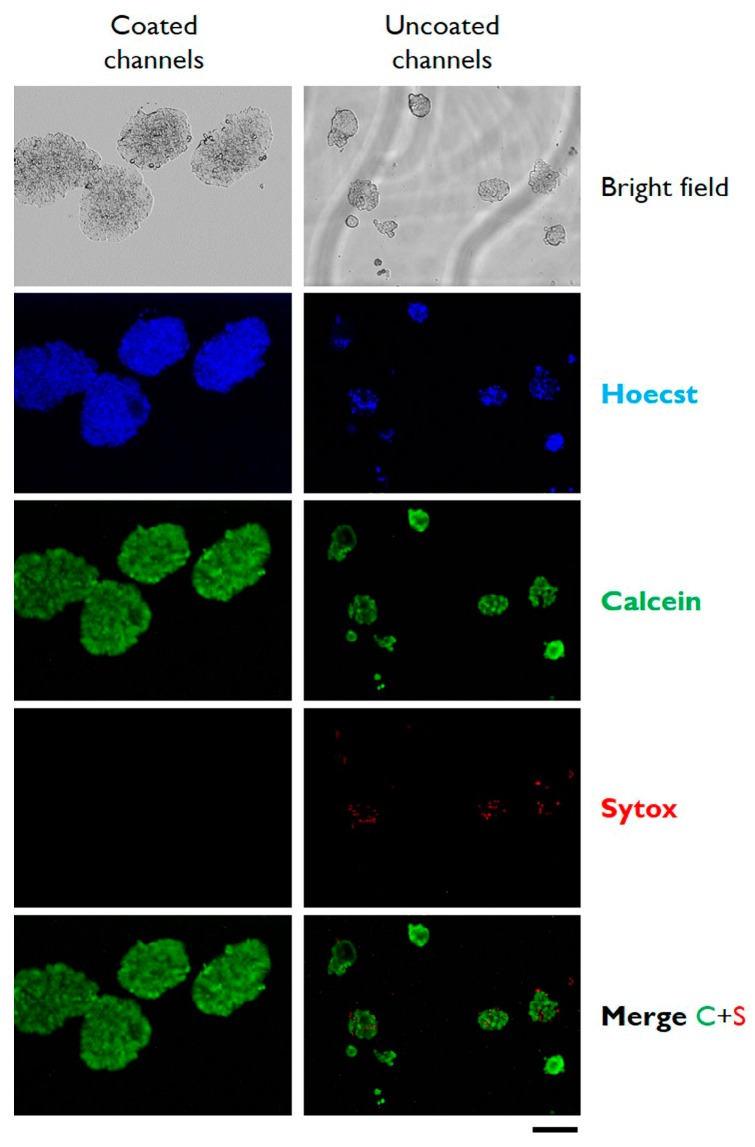
Typical bright field and epifluorescence microphotographs of HT29 cells cultured in gelatin-coated microchannels (left column) or in control uncoated microchannels (right column). Images were taken after 7 days of in vitro cell culture. Cells were stained with the indicated fluorescence dyes before microscopic analysis. Scale bar: 200 µm. The depicted experiment was carried out in triplicate.

To characterize the cells growing in microfluidic conditions, cell viability was determined at day 4 from seeding by live/dead cells stained with Calcein AM and Sytox. In addition, cells were imaged by epifluorescence microscopy after staining with Hoecst. As reported in Figure 3, cells growing as 3D microtissues in microfluidic were highly viable without any detectable signals of dead cells stained with sytox. Interestingly, the cells cultured in the same microfluidic conditions on uncoated microchannels display low but detectable levels of dead cells. The live/dead cell staining was conducted also for HT 29 in multiwells; pictures of Figure S2 confirm the very high viability (almost 100%) of the cells cultured on gelatin dehydrated films. Similar results of aggregation and 3D microtissue formation were obtained using a different cell line, the HepG2 cells (Figure S3).

**Figure 4:**
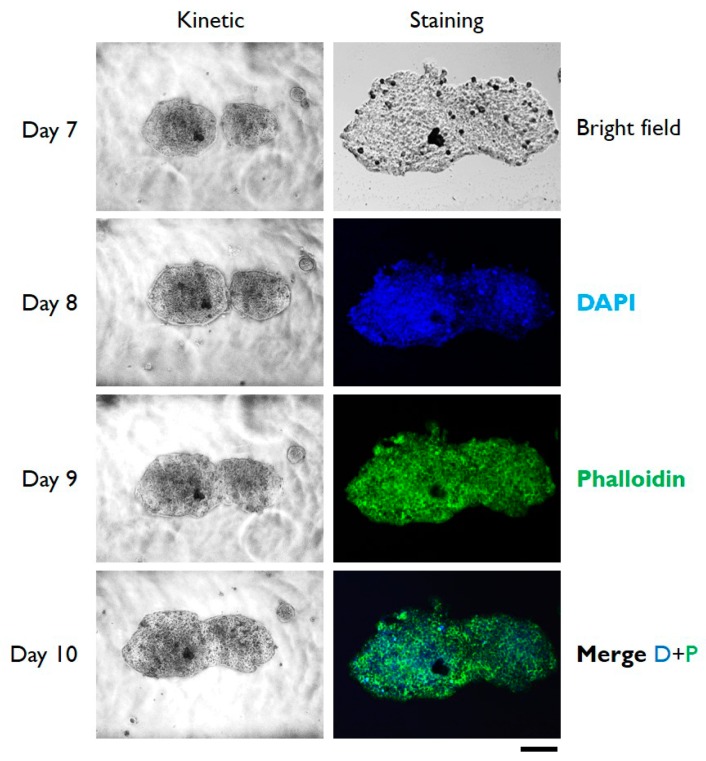
Typical bright field and epifluorescence microphotographs of HT29 cells cultured in gelatin-coated microchannels. Images of the left column show the kinetic of growing of 3D microtissues from days 7 to 10 of in vitro cell culture. The right column shows the confocal fluorescence images taken after 10 days of culture with cells staining with the indicated dyes. Scale bar: 200 µm. The depicted experiment was carried out in triplicate.

In order to verify if cells growing on microchip can be cultured for long period, HT29 cells were analyzed up to 10 days from seeding on-chip. Data reported in Figure 4 indicate that not only the cells continued to proliferate, resulting in 3D microtissues of larger dimensions (see data reported in Table 1) compared to day 7, but also the cells remained highly viable and structurally sound, as proved by the confocal microscopic analysis reported in the right column of Figure 4.

**Figure 5:**
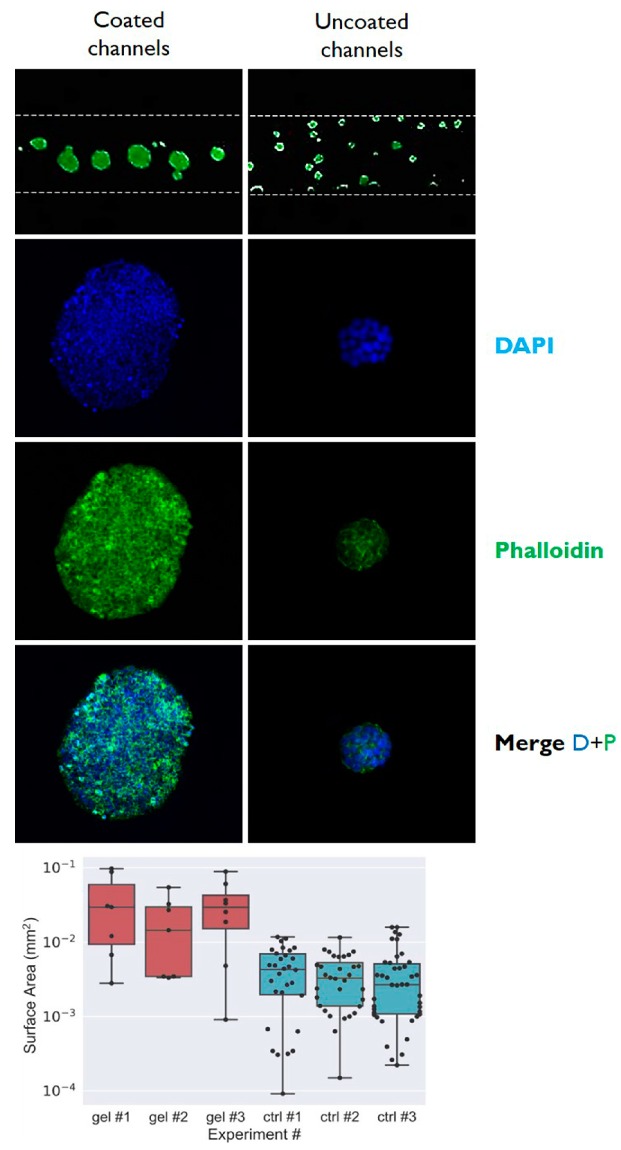
Confocal microscopic analysis of 3D microtissues produced in gelatin-coated microchannels (left column) and in control uncoated microchannels (right column). The histogram reports the mean dimensions (n = 5) (expressed as surface area in mm^2^) of 3D microtissues obtained by HT29 cell culture in the gelatin-coated microchannels (red bars) and in control uncoated microchannels (blue bars) after 5 days of cell culture. Dots represent individual data points, boxes are the quartiles of the dataset, horizontal lines are the mean values, and top and bottom horizontal lines represent the min and max values.

To analyze the differences in dimension of cell aggregates obtained in microfluidic conditions with or without gelatin coating, the surface area of the 3D microtissues were determined by ImageJ analysis after 5 days of cell culture from seeding. The determination reported in the histogram of Figure 5 indicates that the surface area (expressed in mm^2^) of 3D microtissues obtained in coated microchannels was 0.04 mm^2^, whilst that of microtissue obtained in uncoated microchannel was about 10 times lower. Dots represent individual data points, boxes are the quartiles of the dataset, horizontal lines are the mean values, and top and bottom horizontal lines represent the min and max values. In addition, the different dimensions of the obtained microtissues are evident from the top view of the microchannels that are reported in the top images of Figure 5.

**Figure 6:**
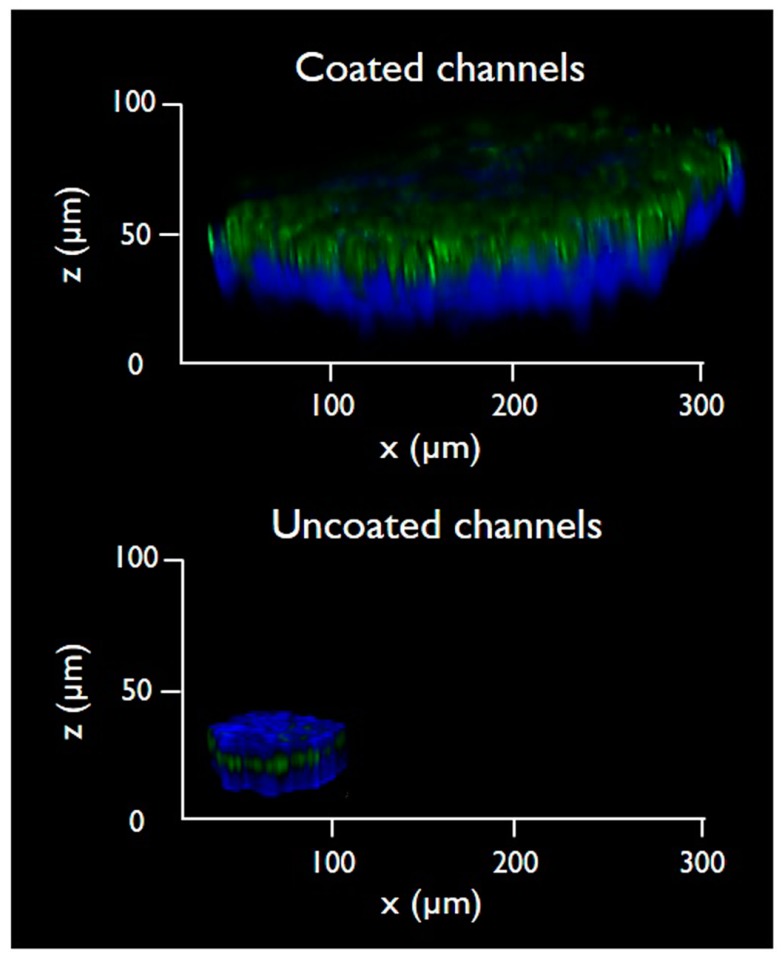
Three-dimensional analysis of 3D microtissues produced in gelatin-coated microchannels (top) and in control uncoated microchannels (bottom). The 3D reconstruction is the projection along z-axis of confocal z-sectioning images (z-slice thickness = 5.28 μm). The depicted experiment was carried out in triplicate.

To further characterize the dimensions of 3D microtissues obtained in microfluidic conditions, the cells were analyzed by confocal laser microscopy to acquire Z-stacks and to reconstruct the 3D structure of the cell cultivated on-chip (Figure 6). The samples were fixed on-chip and stained with DAPI and Alexa Fluor™ 488 Phalloidin. The 3D reconstruction of the microtissues was performed using the LAS X Life Science microscope software, and the cell density was analyzed. The microtissue formed on the gelatin-coated microchannel appeared to have a denser structure when compared to the control cells.

3D microtissues obtained in microfluidic with gelatin-coated microchannels are not only larger in term of surface area (as reported in Figure 5) but they are also substantially thicker with an average thickness of about 100 µm. It is evident that control cells grown on uncoated microchannels have a more flattened morphology, with a thickness below 30 µm.

## 4. Conclusions

A wide range of hydrogel-based 3D cell culture technologies have been developed to address the need for improving cell-based assay as a tool for drug discovery and screening. Most of these methods rely on the use of costly biomaterials; therefore, in this paper, we have explored the possibility to use the alternative and versatile gelatin to coat microfluidic channels and multi-well plates (as control). We demonstrated the applicability of dehydrated gelatin films to fabricate ultra-low attachment microfluidic channels for 3D microtissue formation. The described method, using the commercially available gelatin, turned out to be much cheaper than other film-forming hydrogels constituted of type I collagen, hyaluronic acid, or Matrigel. In addition, the herein-proposed approach allows one to tune the thickness and morphology of the films by adjusting the spin coating conditions. Finally, the method does not use chemical cross-linkers that are often toxic to cells, suggesting that gelatin-coated microchannels could be used for microscale applications, including the creation of tumor models for on-chip drug testing.

## Figures and Tables

**Table 1:** Kinetic of dimensional growth of 3D microtissues cultured in gelatin-coated and uncoated microfluidic chips.

Day	Semi-Major Axis Coated (µm)	Semi-Minor Axis Coated (µm)	Semi-Major Axis Uncoated (µm)	Semi-Minor Axis Uncoated (µm)	Total Area Coated (mm^2^)	Total Area Uncoated (mm^2^)
**7**	201 ± 20	153 ± 12	50 ± 10	41 ± 7	0.965	0.064
**8**	216 ± 24	162 ± 18	53 ± 8	43 ± 4	1.098	0.071
**9**	249 ± 18	165 ± 26	60 ± 12	47 ± 9	1.290	0.088
**10**	262 ± 24	172 ± 9	72 ± 9	54 ± 11	1.415	0.122

Data represent the average dimensions of microtissues from three independent experiments ± SD; for each experiment, 10 microtissues for each condition were measured.

## Supplementary Materials

The following are available online at https://www.mdpi.com/2072-666X/10/4/265/s1, Figure S1: Microphotographs of HT29 cells cultured in gelatin coated (left column) or in control uncoated (right column) multiwells. Images were taken immediately after the seeding (day 0) and after the indicated length of in vitro cell culture. Scale bar 200 µm. Figure S2: Bright field and epifluorescence microphotographs of HT29 cells cultured in gelatin coated (left column) or in control uncoated (right column) multiwells. Images were taken after 4 days of in vitro cell culture. Cells were stained with the indicated fluorescence dyes before microscopic analysis. The graph reports the total number of cells during the cell seeding. Scale bar 200 µm. Figure S3: Microphotographs of HepG2 cells cultured in gelatin coated (left column) or in control uncoated (right column). Images were taken immediately after the seeding (day 0) and after the indicated length of in vitro cell culture. Scale bar 200 µm.
