# Supplementary material for: Gelatin-Coated Microfluidic Channels for 3D Microtissue Formation: On-Chip Production and Characterization
Source: Micromachines (Basel). 2019 Apr 19;10(4):265. doi: 10.3390/mi10040265 (PMC6523541; doi:10.3390/mi10040265)
Supplement: Supplementary file 1 [file micromachines-10-00265-s001.pdf]

micromachines

MDPI

Communication

Gelatin-Coated Microfluidic Channels for 3D Microtissue Formation: On-Chip Production and Characterization

Gabriele Pitingolo [1,*,†], Antoine Riaud [1], Claudio Nastruzzi [2] and Valerie Taly [1,*]

[1] INSERM UMR-S1147, CNRS SNC5014, Paris Descartes University, 75005 Paris, France. Equipe labellisée Ligue Nationale contre le cancer. antoine.riaud@gmail.com

[2] Dipartimento di Scienze Chimiche e Farmaceutiche, Università di Ferrara, 44121 Ferrara, Italia; nas@unife.it

† Present address: Département de Chimie, UMR 8640 PASTEUR, Ecole Normale Supérieure-PSL Research University, 75005 Paris, France.

* Correspondence: gabriele.pitingolo@ens.fr (G.P.); valerie.taly@parisdescartes.fr (V.T.)

Received: 19 March 2019; Accepted: 9 April 2019; Published: 19 April 2019

Abstract. This supplementary material provides all the additional information and a more detailed discussion of the current study.

Coating with Gelatin Film in Multiwells

The 6-well plate were coated with the thin dehydrated gelatin film. The process was optimized to obtain a uniform and thin layer, pouring evenly in each well 300 µL of liquid gelatin dispersion (15% w/v) pre-heated at 45–50 °C. To cover the total area of the well and obtain a uniform layer, the cell culture plate was flipped vertically. After few minutes allowing the stabilization of the liquid gelatin, 6-well plates were placed at 4 °C for 5 min to finalize the gelling process. After gelling, the gelatin-coated 6-well plates were placed at room temperature and then dried at 24–25 °C for 36 h where the gel sample dried slowly but uniformly without deformations on the surface. For the 6-well plate, 1 mL of the cell suspension (0, 3 × 10^6 cells/mL) was added in each well containing 3 mL of medium.

Tumor cells counting protocol

300,000 HT-29 cells were seeded in 3 mL of medium in wells (6-well plate). After 2 and 4 days of cultivation, the total number of cells in each well was analysed using a Countess II FL Automated Cell Counter. 500 µL of Trypsin (0.05%) was added in each well to re-suspend the 3D microtissues (coated) and the cell colonies (uncoated). The cell count was performed by injecting 10 µL of cell suspensions into the counting chamber of the Automated Cell Counter.

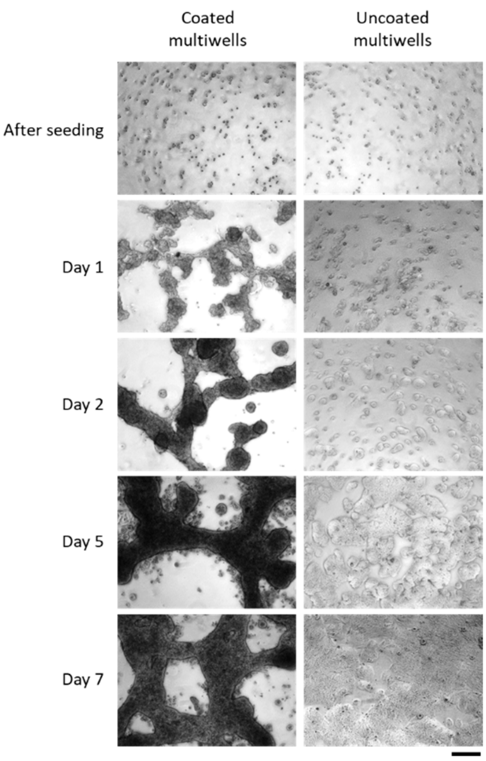

Figure S1. Microphotographs of HT29 cells cultured in gelatin coated (left column) or in control uncoated (right column) multiwells. Images were taken immediately after the seeding (day 0) and after the indicated length of in vitro cell culture. Scale bar 200 μm.

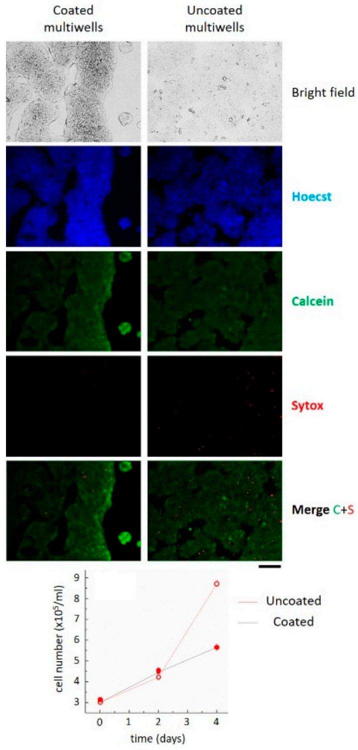

Figure S2. Bright field and epifluorescence microphotographs of HT29 cells cultured in gelatin coated (left column) or in control uncoated (right coloumn) multiwells. Images were taken after 4 days of in vitro cell culture. Cells were stained with the indicated fluorescence dyes before microscopic analysis. The graph reports the total number of cells during the cell seeding. Scale bar 200 μm.

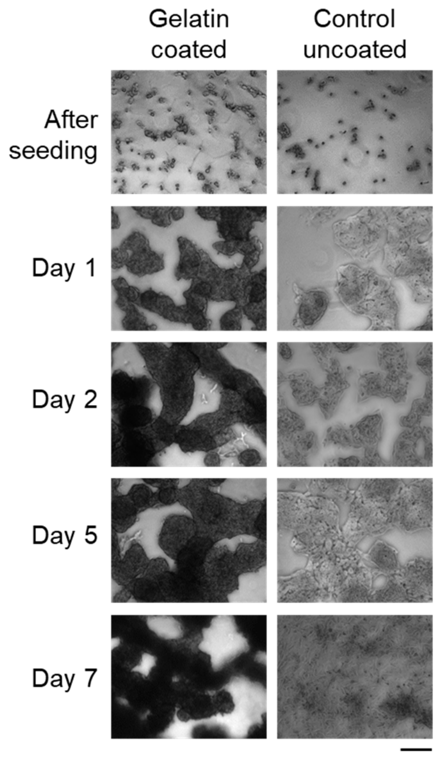

Figure S3. Microphotographs of HepG2 cells cultured in gelatin coated (left column) or in control uncoated (right column). Images were taken immediately after the seeding (day 0) and after the indicated length of in vitro cell culture. Scale bar 200 μm.

© 2019 by the authors. Submitted for possible open access publication under the terms and conditions of the Creative Commons Attribution (CC BY) license (http://creativecommons.org/licenses/by/4.0/).
